# Sex Differences in the Interaction of Epigenetic Risk and Trajectories of Default Mode Limbic Network Integration Predicting Childhood Anxiety

**DOI:** 10.21203/rs.3.rs-9286569/v1

**Published:** 2026-04-19

**Authors:** Kendall Parks, Jessica Uy, Jessica Buthmann, Xinyan Tao, Ai Peng Tan, Ian Gotlib

**Affiliations:** Stanford University; Stanford University; Stanford University

**Keywords:** Epigenetics, DNA methylation, functional brain networks, childhood anxiety, sex differences

## Abstract

**Background:**

Anxiety symptoms often emerge in early development and are more prevalent in females than in males. DNA methylation (DNAm) of stress-related genes at birth may encode susceptibility for anxiety; however, effects may depend on sex and maturation of neural systems involved in self-reference (default mode network; DMN) and attentional-emotional processing (dorsal attention network; DAN). We examined whether DNAm at birth interacts with sex and trajectories of DMN-limbic and DAN-limbic network integration to predict anxiety in early adolescence.

**Methods:**

Participants were children from the Growing Up in Singapore Towards healthy Outcomes (GUSTO) study (N = 97). DNAm was assayed from cord blood (126 CpG sites) and reduced using principal component analysis. Network integration was derived from resting-state fMRI data at ages 4.5, 6, 7.5, and 10.5 years. Trajectories of DMN-limbic and DAN-limbic network integration were estimated using mixed-effects modeling. Anxiety symptoms were assessed at 13 years using the Multidimensional Anxiety Scale for Children. Regression models tested interactions among DNAm, sex, and trajectories of network integration predicting anxiety.

**Results:**

A significant three-way interaction of DNAm, sex, and trajectories of DMN-limbic network integration predicted anxiety symptoms (B = 0.56, SE = 0.24, p=.022). Greater DNAm at birth predicted lower anxiety in males with slower increases in DMN-limbic network integration (B=−0.16, SE = 0.25, p=.021). No significant associations were found for females at any level of network integration or for males with faster increases in DMNlimbic network integration. DAN-limbic network integration had no moderation effects.

**Conclusions:**

Sex-specific interactions with DNAm at birth and DMN-limbic network integration trajectories may confer protection against anxiety in early adolescence.

## Introduction

Anxiety disorders are among the most prevalent mental disorders worldwide, contributing to significant long-term distress and health burden (*2021 Global Burden of Disease (GBD)*, 2024; World Health Organization, 2025). Symptoms of anxiety include persistent worrying, restlessness, irritability, difficulty regulating threat-related emotions, and somatic hyperarousal (American Psychiatric Association, 2022; Ramsawh et al., 2010) that interfere with individuals’ daily functioning and quality of life. Among adults, women experience anxiety disorders at twice the rate that men do (Baxter et al., 2013; Mishra & Varma, 2023), a disparity that highlights the importance of examining sex differences in developmental pathways to anxiety. Although anxiety disorders are most often diagnosed in adolescence and early adulthood, symptoms of anxiety can manifest earlier in development (Finsaas et al., 2018; Pollock et al., 1995), underscoring the importance of identifying factors in infancy and early childhood that predict risk for anxiety in late adolescence and adulthood in order to inform the development of effective prevention strategies.

Given the early emergence of anxiety symptoms, it is likely that processes during the perinatal period and early childhood contribute to risk for anxiety. In this context, epigenetic modification, such as DNA methylation (DNAm), is a process in which early susceptibility to anxiety may be biologically embedded. In DNAm, a methyl group is added onto the cytosine-guanine dinucleotide (CpG) sites of a DNA sequence, generating an epigenetic signature that regulates gene transcription (Klengel et al., 2014; Moore et al., 2013). Although patterns of DNAm can be modified over the lifespan in response to environmental factors, they are established primarily during prenatal development and tend to remain relatively stable (Klengel et al., 2014; Reik, 2007); thus, DNAm at birth may be an early biological indicator of subsequent neural and emotional functioning (Abrishamcar et al., 2024; Di Paolo et al., 2025). For example, in a study comparing rats bred for high versus low anxiety, McCoy et al. (2019) found that at postnatal day 7, high-anxiety rats had higher global DNAm and differentially methylated regions in the amygdala, which in turn were associated with anxiety-like behaviors exhibited in adulthood. In humans, few researchers have examined the relation between DNAm at birth and later anxiety outcomes. Pilkay et al. (2024) found that DNAm of genes involved in neural development and oxidative stress regulation in newborns was associated with separation anxiety and fear-related behaviors at age 7, with stronger associations in boys than in girls.

Another key pathway by which early epigenetic modifications might influence the development of anxiety involves stress-related genes. For example, increased methylation of the glucocorticoid receptor gene (*NR3C1* promoter) has been associated with hypothalamus-pituitary-adrenal (HPA)-axis hyperactivity in both adults with generalized anxiety disorder (W. Wang et al., 2017) and adolescents with steeper trajectories of internalizing symptoms (van der Knaap et al., 2015). These findings suggest that DNAm of stress-related genes indexes early variation in patterns of stress-responsive gene expression that are relevant to risk for anxiety. Importantly, associations between DNAm at birth and later anxiety outcomes are likely not uniform across individuals. Instead, early epigenetic variation may confer differential susceptibility to later anxiety, such that the strength and direction of these associations may depend on the development of neural systems involved in emotion processing and regulation across childhood.

In this context, researchers have implicated large-scale brain networks involved in emotional and attentional functioning in anxiety disorders. In particular, alterations in connectivity between and within the default mode network (DMN), which supports self-referential processing, the dorsal attention network (DAN), which supports goal-directed attentional control, and the limbic network, as defined by Yeo et al. (2011) and involved in affective processing and emotional evaluation, have been associated with anxiety primarily in adult samples (De la Peña-Arteaga et al., 2024; Farrant & Uddin, 2015; Karl et al., 2024; Zidda et al., 2018). Most research has used functional connectivity – the strength of communication between pairs of regions – to examine associations among networks. In contrast, graph theoretic approaches extend pairwise connectivity by characterizing how brain regions are organized at the network level into larger functional groupings across the brain (Sporns, 2013). From this perspective, network integration reflects the extent to which regions from different networks are incorporated into shared functional groupings, indexing cross-network coordination at a system level. This network-level organization may be particularly relevant for understanding anxiety, which has been associated with reduced coordination between large-scale brain networks in both adolescents and adults with generalized anxiety disorder (Guo et al., 2021; Leypoldt et al., 2025; Makovac et al., 2018). Research examining network organization in anxiety has largely used cross-sectional designs or single timepoint assessments of brain connectivity, limiting our understanding of how these networks change over the course of development. Examining trajectories of integration between large-scale networks will elucidate how network organization changes across childhood in the context of predicting subsequent levels of anxiety.

Finally, investigators have implicated sex-related differences in neural pathways in the development of anxiety. For example, females with a history of internalizing disorders have been found to have greater decreases in activity in DMN during cognitive interference compared to males with similar histories (Z. Wang et al., 2016). Further, females exhibit greater suppression of the DMN and greater activation of the DAN during attentional processing to emotional content than do males (Dumais et al., 2018). Finally, males and females with internalizing symptoms have been found to be characterized by different patterns of amygdala connectivity: whereas males exhibit hypoconnectivity to prefrontal regions, females show hyperconnectivity to emotion processing regions in the cortex (Padgaonkar et al., 2020). We do not yet know, however, whether males and females differ in how integration between the limbic network and the DMN or DAN is related to anxiety, and whether these sex differences interact with DNAm to affect risk for anxiety in children.

The goal of the current study was to examine the longitudinal effects of DNA methylation at birth, trajectories of DMN- and DAN-limbic network integration from early to middle childhood, and biological sex on anxiety symptoms in early adolescence. Although normative development is characterized by changes in the integration and segregation of large-scale brain networks over childhood (Fair et al., 2008; Menon, 2013), we do not yet know how individual differences in the maturation of network integration are related to the development of anxiety symptoms in early adolescence. We hypothesized that the association between DNAm of stress-related genes at birth and anxiety symptoms in early adolescence will vary as a function of both biological sex and trajectories of self-referential-emotion (DMN-limbic network) and attentional-emotion (DAN-limbic network) integration across childhood. Specifically, we predicted that associations between DNAm and anxiety will be stronger for trajectories of DMN-limbic network integration than for trajectories of DAN-limbic network integration. Further, given the higher prevalence of anxiety in females than in males (Baxter et al., 2013; Mishra & Varma, 2023), combined with sex differences in neural systems involved in emotional processing and attentional regulation (Dumais et al., 2018; Padgaonkar et al., 2020), we also expected that these associations will be stronger in females than in males.

## Methods

### Participants

Participants were drawn from the Growing Up in Singapore Towards healthy Outcomes (GUSTO) birth cohort, a longitudinal study examining perinatal influences on individual differences in neurodevelopment and mental health outcomes (Soh et al., 2014). The study recruited expectant mothers (age 18 years and above) of Chinese, Malay, and Indian descent during their first trimester of pregnancy. Data were collected from the mothers throughout gestation and from their children during structured follow-up visits through 13 years of age. Inclusion criteria required mothers be Singapore citizens or permanent residents who consented to donate umbilical cord blood and tissue for subsequent analyses. Exclusion criteria included < 34 weeks gestational term, admission to the neonatal intensive care unit, non-homogeneous parental ethnic backgrounds, and pre-existing or notable physical and mental health challenges that might confound developmental outcomes (i.e., cancer, cognitive difficulties).

Of the 331 mother-child dyads who provided umbilical cord blood samples at birth, 97 children had at least two good-quality functional magnetic resonance imaging (fMRI) scans available between ages 4.5–10 years and completed a measure of anxiety symptoms at age 13 years. The GUSTO study was approved by both the National Healthcare Group Domain Specific Review Board (Reference #D/09/021) and the SingHealth Centralized Institutional Review Board (IRB) (Reference #20009/280/D). All mothers provided written informed consent prior to engaging in any study activities.

## Measures

### Umbilical cord blood DNA methylation

DNA methylation profiles were generated from umbilical cord blood collected at birth and processed following the procedures described in (Bibikova et al., 2011; Pan et al., 2012). From the 85,624 CpG sites quantified, we focused on 126 *a priori* sites from stress-related genes (Parade et al., 2021). All CpG sites included in the final analysis were located within gene bodies, where higher methylation typically corresponds to increased transcriptional output (Hellman & Chess, 2007). In order to reduce the DNAm features, we used the principal component derived in Parks et al. (2025), which was computed from the beta values of CpG sites after genomic annotation and filtering. Because most of these sites (31 of 36) loaded positively, higher scores on the DNAm component reflected proportionally higher methylation across these loci. CpG sites with the strongest positive loadings mapped onto genes that are central to neuroendocrine and stress-regulatory pathways, including *NCOR2*, *CRHBP*, *HSD11B1*, and *HTR2A*. In contrast, and consistent with their roles in inhibiting stress responses, CpG sites within *ADRA1B*, *CREBBP*, and *NR3C1* had negative loadings. Considered collectively, given the comparatively stronger positive loadings of the CpG sites in *NCOR2*, *CRHBP*, *HSD11B1*, and *HTR2A*, higher DNAm component values reflect increased methylation and upregulation of gene expression in stress-regulatory pathways.

### Resting-state fMRI data and network integration

Resting-state fMRI data were acquired at ages 4.5, 6, 7.5 and 10.5 years using a 3T Siemens Skyra scanner with a 32-channel head coil. Preprocessing and derivation of network-level metrics followed previously published protocols (Nguyen et al., 2024; Q. Wang et al., 2019), which included motion correction, nuisance signal regression, temporal filtering, and alignment to a standard cortical template. Additional details regarding preprocessing and quality control are described in these papers. Graph theory-based measures of functional network integration were derived from these data in the GUSTO cohort using a network-based parcellation from Yeo et al.’s (2011) seven-network solution, in which the limbic network includes regions such as the orbitofrontal and anterior temporal cortices. Integration coefficients quantify the probability that regions from different networks (i.e., DMN, DAN, limbic network) are assigned to the same functional community across repeated iterations of community detection. Higher values indicate a greater likelihood that regions from different networks operate as a unified system within the broader brain architecture, rather than reflecting pairwise functional connectivity between individual regions. For the present study, we examined DMN-limbic network and DAN-limbic network integration at each timepoint. Longitudinal modeling of network integration is described in the Data Analysis section, below.

### Childhood anxiety symptoms

Child anxiety symptoms were assessed at age 13 years using the Multidimensional Anxiety Scale for Children, Second Edition (MASC-2; March, 2013). The MASC-2 is a wellvalidated 50-item self-report measure of trait anxiety with high test-retest reliability and invariance across gender and age. It assesses multiple domains of childhood anxiety, including physical symptoms, generalized anxiety disorder, harm avoidance, social anxiety, obsessions and compulsions, and separation anxiety/phobias. Each item is rated on a 4-point Likert scale reflecting symptom frequency (0 = never true, 3 = often true). Subscale scores were summed to derive a raw total score, with higher scores indicating greater severity of anxiety (see Table 1).

### Maternal prenatal psychopathology

Maternal symptoms of depression and anxiety were assessed at 26 weeks of gestation (PW26) using the Beck Depression Inventory-II (BDI-II; Beck et al., 1996) and the State-Trait Anxiety Inventory (STAI; Spielberger, 1983), respectively. To generate a composite index of prenatal maternal psychopathology, total scores on the BDI-II and STAI were each z-scored and then averaged. Higher values on this composite score reflect more prenatal maternal depressive and anxious symptoms. We included this composite score as a covariate in all analyses to account for prenatal maternal emotional distress that may be associated with perinatal and later offspring outcomes.

### Data analysis

Statistical analyses were conducted using RStudio (RStudio Team, 2020). Continuous variables (including measures of network integration, mean motion parameters, the DNAm component, and anxiety symptoms) were standardized to facilitate interpretation. Minimal missingness in maternal education (*n* = 1) and prenatal maternal psychopathology (*n* = 6) was addressed by imputing values using the sample mode. Multicollinearity among predictors and covariates was minimal (variance inflation factor values < 1.4).

To estimate individual trajectories of DMN-limbic network and DAN-limbic network integration across ages 4.5 to 10.5 years, we fit latent class mixed models in R using the *lcmm* package (RStudio Team, 2022), which allows for simultaneous estimation of latent classes and individual trajectory parameters. Age (centered at minimum age of 4.48 years) was modeled as a continuous time variable with participant-specific random intercepts and random age slopes, adjusting for mean motion. We compared 1-, 2-, and 3-class solutions. Although we tested models with more classes, solutions beyond two classes showed poor class separation (low entropy) and model instability. The 1-class model was retained based on lower Bayesian Information Criterion (BIC) values and improved class stability relative to the 2-class solution, which yielded a small class (~ 6% of participants). Subject-specific slope estimates representing change in network integration across time were extracted from the 1-class model and standardized for regression analyses.

Using multiple regression analyses, we tested whether the association between DNAm and anxiety symptoms is moderated by trajectories of network integration and biological sex. We conducted separate analyses for DMN-limbic and DAN-limbic network integration. To account for testing two network models, we applied a Bonferroni correction (α = .025). In all models we controlled for child ethnicity, gestational age, maternal age at birth and education levels, and maternal symptoms of depression and anxiety at PW26. Finally, we examined variance inflation factors to assess multicollinearity and visually inspected residual plots to verify that assumptions of normality and homoscedasticity were met.

## Results

### Participant characteristics

Demographic characteristics of the mother-child dyads, along with descriptive statistics for the DNAm component, trajectories of DMN- and DAN-limbic network integration, and anxiety symptoms, are presented in Table 1. On average, trajectories of DMN-limbic network integration were positive (M = 0.8, SD = 0.11), indicating greater functional integration between these networks over the ages of 4.5 to 10.5 years. In contrast, trajectories of DAN-limbic network integration were negative (M = −0.13, SD = 0.05), indicating decreasing integration between the DAN and limbic network over time. Comparison of dyads included (n = 97) versus excluded (n = 234) in the final analytic sample yielded a significant difference in maternal education (*X*^*2*^ = 9.91, *p* = .042): excluded participants were more likely to have secondary education or university-level education, whereas included participants were more evenly distributed across education levels. The two groups did not differ significantly in any other demographic variables (see Supplementary Table S1).

### Sex Differences in the Moderation of Epigenetic Risk for Anxiety by Trajectories of Network Integration

**Figure 1:**
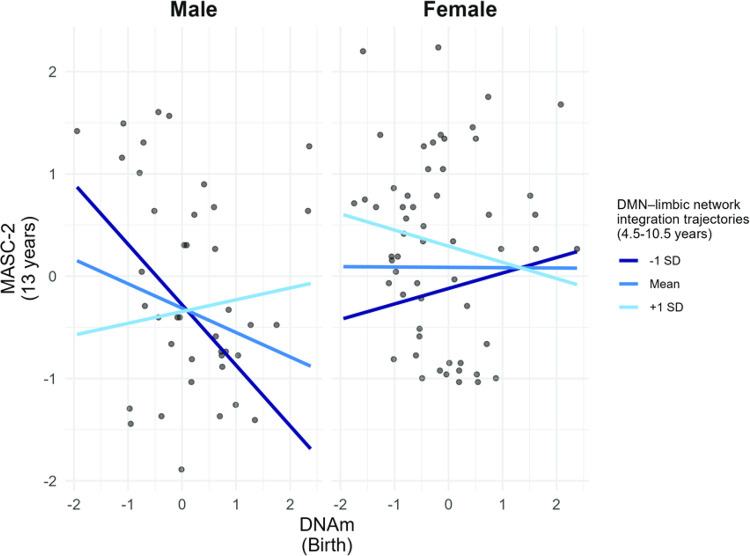
The association between DNA methylation at birth and anxiety symptoms in late childhood as a function of DMN-limbic network integration trajectories and sex

We examined whether the association between DNAm at birth and anxiety symptoms at age 13 differed as a function of biological sex and trajectories of DMN- and DAN-limbic network integration from ages 4.5–10.5 years. There was a significant three-way interaction of DNAm, trajectories of DMN-limbic network integration, and sex (*B* = 0.56, SE = 0.24, *t* = 2.33, *p* = .022; Fig. 1). To interpret this interaction, we conducted simple slope analyses examining the association between DNAm and anxiety separately for boys and girls at different levels of DMN-limbic network integration (see Table 2 and Fig. 1). In males with slower increases in DMN-limbic network integration from ages 4.5–10.5 years, DNAm at birth was negatively associated with anxiety symptoms at age 13 years (*B* = −0.16, SE = 0.25, *t* = −2.35, *p* = .021). DNAm was not associated with anxiety symptoms in females at any level of DMN-limbic network integration (all *p* > .38). For DAN-limbic network integration, the three-way interaction of DNAm, trajectories of DAN-limbic network integration, and sex was not significant (*B* = 0.56, SE = 0.41, *t* = 1.13, *p* = .184), indicating that trajectories of DAN-limbic network integration did not moderate the association between DNAm and anxiety symptoms in adolescence in either sex. See Supplementary Table S2 for the simple slopes for DAN-limbic network integration.

## Discussion

This study was designed to examine whether DNA methylation patterns at birth and DMN-limbic network integration from early to middle childhood interact with biological sex to predict anxiety symptoms at age 13 years. We found a significant three-way interaction of DNAm, trajectories of DMN-limbic network integration, and biological sex predicting subsequent anxiety symptoms. Specifically, among males with slower increases in DMN-limbic network integration over time, greater DNAm of stress-related genes at birth was associated with lower anxiety symptoms at age 13. This association was not significant for females at any level of DMN-limbic network integration or for males with mean or higher levels of network integration. We also examined whether DAN-limbic network integration moderated the association of DNAm and anxiety; however, the three-way interaction of DNAm, DAN-limbic network integration, and sex was not significant. Thus, these findings suggest that the sex-specific epigenetic effects on anxiety we documented in this study are specific to integration between the DMN and limbic network.

The trajectories of DMN-limbic network integration from ages 4.5 to 10.5 years arguably reflect the maturation of cognitive-emotion systems during a sensitive period in brain development. Increases in integration across large-scale networks have been posited to reflect greater coordination among distributed brain systems, rather than changes in the strength of individual connections (Fair et al., 2008; Power et al., 2010). In addition, DMN intrinsic connectivity has been found to be weak between the ages of 7 and 9 years and to increase with age, supporting the specialization of self-referential processing (Fair et al., 2008; Sato et al., 2014). Thus, the slope of DMN-limbic network integration across this period may index individual differences in the development of coordination between self-referential and affective neural systems. The specificity of effects to DMN- rather than DAN-limbic network integration suggests that self-referential and internally directed emotional processing, rather than top-down attentional control systems, are important in determining how epigenetic factors influence the development of anxiety. Although both networks have been associated with anxiety symptoms, the DMN has been found to support self-focused worry and rumination that characterize anxiety disorders (Steinfurth et al., 2017; Zhou et al., 2020). Goal-oriented attentional control processes associated with the DAN may be less central to the expression of anxiety symptoms (Abend et al., 2020; Farrant & Uddin, 2015) and may operate through different developmental pathways that are less sensitive to early epigenetic programming.

Our findings are consistent with prior work documenting altered DMN-limbic network connectivity in anxiety disorders. Lower levels of functional connectivity between DMN regions and limbic structures have been associated with higher trait anxiety in both adults and youth. For example, Zidda et al. (2018) found that stronger connectivity between DMN regions and limbic structures (amygdala and hippocampus) was associated with better fear and anxiety learning in healthy volunteers. Similarly, Chen and Etkin (2013) found that connectivity between the posterior hippocampus and DMN is disrupted in anxiety-related disorders. Extending this work, graph theoretic studies have shown that anxiety is characterized by reduced coordination between large-scale networks. For example, Guo et al. (2021) reported decreased inter-network connectivity in adolescents with generalized anxiety disorder, reflecting reduced coordination among large-scale systems that included higher-order cortical networks such as the DMN. Consistent with this finding, reduced global network efficiency was also reported in adults with generalized anxiety disorder (Makovac et al., 2018). In contrast, our findings suggest that the developmental pattern of network integration has different implications for anxiety than do differences observed in clinical samples in cross-sectional studies. Slower increases in DMN-limbic integration during this period may reflect more gradual coordination between self-referential and affective systems, which may be protective in specific biological contexts. The current findings extend prior cross-sectional work by demonstrating that developmental trajectories of network integration interact with early biological risk factors to predict anxiety outcomes in a sex-specific manner.

Contrary to our hypothesis, we found that the interaction between DNAm and trajectories of DMN-limbic network integration was significant in males but not in females. Thus, sex differences in the association between DNAm and anxiety do not seem to be explained solely by differences in disorder prevalence, but instead, depend on how early epigenetic variation is related to patterns of neural development. The role of sex in this association may reflect one or more of two underlying mechanisms. First, sex differences may be related to differences in stress-responsive biological systems. Prenatal maternal stress has been found to be associated with stronger HPA-axis reactivity in female than in male offspring (Carpenter et al., 2017; McCormick et al., 1995), indicating that these systems function differently in males and females early in development. Given that our DNAm component includes genes involved in HPA-axis function and stress-responsive processes (e.g., *NCOR2, CRHBP*, *HSD11B1*), sex-specific calibration of these systems may be reflected in differential methylation patterns of these genes at birth. Second, sex differences in early neurodevelopmental organization may contribute to the observed pattern. Gonadal steroids during early brain development have been associated with sex-specific patterns of neural regulation and stress responsivity that may persist throughout development (Patchev & Almeida, 1998), suggesting that early hormonal influences on neural organization contribute to sex differences in the relation of epigenetic variations and the development of neural systems involved in anxiety.

The finding that the effects of DNAm on anxiety symptoms are stronger in males with specific patterns of neural network integration suggests that early epigenetic modifications interact with neural development in predicting emotional outcomes. DNAm is a key mechanism for biological embedding, with prenatal and perinatal factors associated with lasting effects on brain function and behavior (Klengel et al., 2014; Moore et al., 2013). The stress-related genes included in our DNAm component—such as *NCOR2, CRHBP, HSD11B1, and HTR2A*—are involved in neuroendocrine and stress-regulatory pathways, suggesting that increased methylation patterns at these gene body loci are associated with the early calibration of stress response systems. By modulating gene expression, these epigenetic signatures may influence the development of specific neural circuits, including those connecting prefrontal and limbic regions that are critical for emotional regulation and stress responsiveness. Notably, we found the interaction of higher DNAm of stress-related genes and slower increases of DMN-limbic network integration to be associated with lower levels of anxiety symptoms in males, suggesting that specific combinations of epigenetic and neural factors confer protection against the development of anxiety symptoms in early adolescence. One interpretation of this finding is that slower integration between self-referential (DMN) and affective systems reflects reduced coordination of internal thought and emotional processing. This reduced coordination may limit the amplification of negative affect, consistent with findings that stronger functional coupling between these systems is associated with increased rumination (Hamilton et al., 2015; Ho et al., 2015). This finding highlights the importance of developmental timing for intervention approaches that target self-referential processing and emotional regulation in early childhood. For example, mindfulness-based practices (Tymofiyeva et al., 2021) or cognitive-behavioral programs such as the Coping Cat intervention (McNally Keehn et al., 2013) may support coordination between these systems. Further, recognizing that specific epigenetic profiles can be protective rather than harmful in particular neural contexts challenges traditional risk-focused approaches and underscores the importance of nurturing developmental conditions that can help maintain these protective effects.

We should note four limitations of this study. First, our sample size was relatively small, which may limit generalizability and statistical power. Future studies with larger samples are needed to test the reliability of the three-way interaction reported here. Second, while our sample represents multiple ethnic groups and varying socioeconomic levels in the urban Singaporean context, replication across other populations and cultures would strengthen these findings. Third, we examined anxiety symptoms at a single time point (age 13 years). Future studies should examine these associations across other developmental periods. Finally, we did not assess paternal or second parent psychopathology, parent-child interactions, or broader family environment factors that may influence neural development and anxiety outcomes. Future research should examine how family-level factors, such as family support systems and parenting behaviors, contribute to these observed interactions.

Despite these limitations, a key strength of this study is the longitudinal design that captured developmental trajectories of network integration across a critical period of large-scale neural network maturation. We also integrated epigenetic, neural, and behavioral measures to provide a multimodal perspective on developmental pathways for anxiety. Finally, our examination of sex differences revealed an epigenetic-brain interaction that was specific to males, a finding that would have been missed in sexcombined analyses. This study advances our understanding of sex-specific developmental associations among early epigenetic signatures, the maturation of neural network integration, and later anxiety outcomes. Future research building on our findings may help inform targeted prevention and intervention efforts during critical periods of development.

## Supplementary Material

Supplementary Files

This is a list of supplementary files associated with this preprint. Click to download.

- ParksGUSTODMNLimbicSuppMat.docx

## Figures and Tables

**Table 1:** Characteristics of the mother-child dyads

Infant	n = 97		%
		
**Sex**			
Female	57		58.8
Male	40		41.2
**Gestational age**			
<37 weeks	7		7.2
37–40 weeks	72		74.2
>40 weeks	18		18.6
**Mother**			
**Age**			
18–24 years	14		14.4
25–34 years	61		62.9
35–44 years	22		22.7
**Ethnicity**		
Chinese	55		56.7
Malay	27		27.8
Indian	15		15.5
**Highest education level**			
Primary/elementary	11		11.3
Secondary/high school	19		19.6
Vocational/ITE/NITEC	16		16.5
A levels/polytechnic/diploma	29		29.9
University or higher	22		22.7
**Maternal Psychopathology (PW26)**			
BDI-II	Mean: 9.74	SD: 7.21	Range: 0–39
STAI	Mean: 36.70	SD: 9.93	Range: 20–61
**Variables of Interest**			
DMN-limbic network integration (slope)	Mean: 0.80	SD: 0.11	Range: 0.38–1.09
DAN-limbic network integration (slope)	Mean: −0.13	SD: 0.05	Range: −0.26–0.05
DNAm component	Mean: −0.15	SD: 0.93	Range: −2.07–2.24
MASC	Mean: 60.31	SD: 25.58	Range: 6–117

Note: DAN = Dorsal Attention Network; DMN = Default Mode Network; DNAm = DNA methylation; MASC = Multidimensional Anxiety Scale for Children; PW26 = Pregnancy Week 26.

**Table 2:** Sex-specific simple slopes for the association between DNAm at birth and anxiety symptoms across levels of DMN-limbic network integration

Sex	DMN-Limbic network integration	*B*	SE	T	p
**Females**	Low (−1 SD)	0.15	0.17	0.88	.381
	Mean	−0.01	0.14	−0.02	.984
	High (+ 1 SD)	−0.16	0.19	−0.82	.415
**Males**	Low (−1 SD)	−0.16	0.25	−2.35	.021
	Mean	−0.24	0.16	−1.54	.127
	High (+ 1 SD)	0.12	0.21	0.54	.591
